# A strategy for the selection of monovalent antibodies that span protein dimer interfaces

**DOI:** 10.1074/jbc.RA119.009213

**Published:** 2019-08-06

**Authors:** Jamie B. Spangler, Ignacio Moraga, Kevin M. Jude, Christina S. Savvides, K. Christopher Garcia

**Affiliations:** ‡ Howard Hughes Medical Institute, Stanford University School of Medicine, Stanford, California 94305; § Department of Molecular and Cellular Physiology, Stanford University School of Medicine, Stanford, California 94305; ¶ Department of Structural Biology, Stanford University School of Medicine, Stanford, California 94305; ‖ Department of Biomedical Engineering, Johns Hopkins University, Baltimore, Maryland 21218; ** Department of Chemical and Biomolecular Engineering, Johns Hopkins University, Baltimore, Maryland 21218; ‡‡ Department of Biology, Stanford University School of Medicine, Stanford, California 94305

**Keywords:** antibody engineering, dimerization, cytokine, immunology, structural biology, directed evolution, interleukin-2, interleukin-4, ligand-receptor interactions, cell signaling

## Abstract

Ligand-induced dimerization is the predominant mechanism through which secreted proteins activate cell surface receptors to transmit essential biological signals. Cytokines are a large class of soluble proteins that dimerize transmembrane receptors into precise signaling topologies, but there is a need for alternative, engineerable ligand scaffolds that specifically recognize and stabilize these protein interactions. Recombinant antibodies can potentially serve as robust and versatile platforms for cytokine complex stabilization, and their specificity allows for tunable modulation of dimerization equilibrium. Here, we devised an evolutionary strategy to isolate monovalent antibody fragments that bridge together two different receptor subunits in a cytokine–receptor complex, precisely as the receptors are disposed in their natural signaling orientations. To do this, we screened a naive antibody library against a stabilized ligand–receptor ternary complex that acted as a “molecular cast” of the natural receptor dimer conformation. Our selections elicited “stapler” single-chain variable fragments (scFvs) of antibodies that specifically engage the interleukin-4 receptor heterodimer. The 3.1 Å resolution crystal structure of one such stapler revealed that, as intended, this scFv recognizes a composite epitope between the two receptors as they are positioned in the complex. Extending our approach, we evolved a stapler scFv that specifically binds to and stabilizes the interface between the interleukin-2 cytokine and one of its receptor subunits, leading to a 15-fold enhancement in interaction affinity. This demonstration that scFvs can be selected to recognize epitopes that span protein interfaces presents new opportunities to engineer structurally defined antibodies for a broad range of research and therapeutic applications.

## Introduction

**Figure 1.**
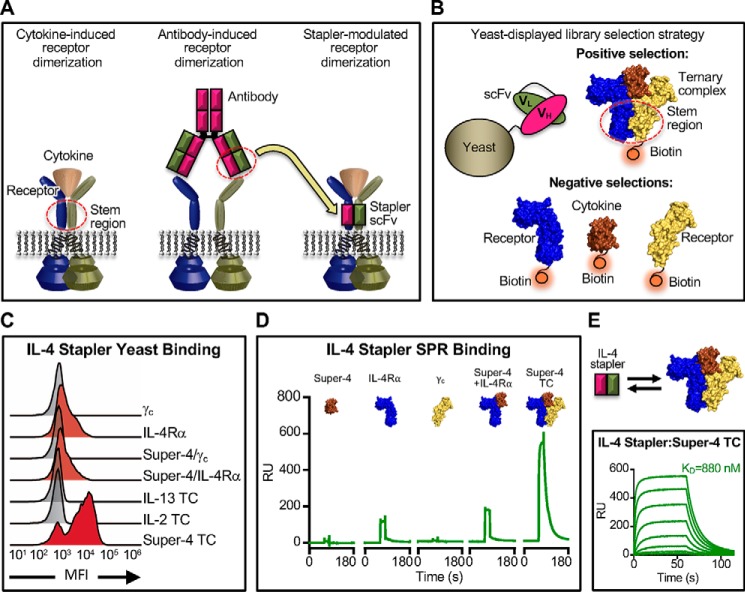
**Novel evolutionary selection strategy isolates antibody-based “staplers” that dimerize cytokine receptor chains.** *A*, *schematic* contrasting receptor heterodimerization and activation induced by cytokine, antibody, or “stapler” scFv binding, respectively. *B*, layout and selection scheme for yeast display-based evolution of cytokine–receptor complex staplers. A yeast-displayed scFv library is evolved through iterative rounds of selection, each of which comprises negative clearance steps against the individual complex components and positive selection for binders to the fully assembled ternary complex. *C*, flow cytometry histograms depicting the binding of yeast-displayed IL-4 stapler scFv to the indicated cytokine–receptor complexes and individual components thereof. *TC*, ternary complex. *D*, kinetic SPR binding profiles for the interactions between the immobilized IL-4 stapler scFv and soluble Super-4/IL-4Rα/γ_c_ TC components *versus* the fully assembled complex. All proteins were flowed at a concentration of 60 μm. *E*, surface plasmon resonance kinetic binding traces depicting the interaction between immobilized IL-4 stapler scFv and dilutions of the Super-4/Il-4Rα/γ_c_ ternary complex. The equilibrium dissociation constant (*K_D_*) value was calculated by fitting the equilibrium titration curve to a logistic model via nonlinear regression. The *top curve* represents a concentration of 20 μm, and *subsequent curves* represent 3-fold serial dilutions.

Ligand-mediated receptor dimerization is the most prevalent signaling mechanism used by secreted proteins to activate their cognate cell surface receptors. In particular, transmembrane receptors of the cytokine and receptor tyrosine kinase families signal when oriented into specific receptor dimer geometries (1–3). Cytokines constitute a class of soluble ligands that act through dimeric membrane-embedded receptors to elicit a wide range of biological activities, particularly those pertinent to immune regulation (3–5). Cytokines bind to receptor extracellular domains (ECDs)^4^ and either reorient quiescent dimers or enforce dimerization of monomeric subunits (Fig. 1*A*, *left*) (6–11). Extracellular dimerization results in activation of Janus kinases (JAKs) constitutively associated with the receptor intracellular domains, which recruit and activate signal transducer and activator of transcription (STAT) proteins to effect transcriptional programs that determine cell fate (12–15).

There has been a great deal of interest in harnessing the agonistic potential of JAK/STAT cytokines as immunotherapeutics, but so far success has been very limited for several practical reasons (16, 17). First, the short *in vivo* half-life (typically <5 min) of cytokines mandates frequent injection or continuous infusion. Second, cytokines are pleiotropic, often activating a wide range of cell types expressing shared receptors, which hinders efficacy and can lead to systemic toxicity. Finally, these ligands are difficult to re-engineer or modify without concerns about immunogenicity (18–22). Thus, there exists a need for new modulators of protein dimerization that are based on protein scaffolds with both improved druglike properties and the capacity to serve as engineering substrates.

Monoclonal antibodies present stable, engineerable scaffolds that benefit from extended *in vivo* half-life due to interactions with neonatal Fc receptors (23, 24), and they can act as bivalent dimerization modulators for cytokine receptors. Previous work has demonstrated that certain cytokine receptor-targeted bivalent antibodies can activate signaling in the absence of cytokine (Fig. 1*A*, *center*) (25–31). However, these molecules enforce unnatural receptor dimer geometries, which can impact the nature of signal activation (Fig. 1*A*, *center*) (31). Antibodies that potentiate cytokine action by enhancing cytokine–receptor binding have also been reported (32, 33). Here, we sought to develop an evolutionary strategy for the discovery of antibody-based constructs that simultaneously engage two interacting subunits within dimerized receptors, as they are disposed in their native signaling conformations induced by endogenous cytokines (Fig. 1*A*, *right*).

Antibody Fab fragments or single-chain variable fragments (scFvs) are widely known to recognize “composite” ligand surfaces, such as Fabs that bind across the interfaces between different subunits of a multimeric antigen. Yet it is not clear whether such composite interface-recognizing Fabs can be evolved to specifically recognize and bridge protein interfaces. We wished to isolate monovalent antibodies (which we term “staplers”) that bind to the composite dimer surface formed by the “stem region” of the receptors when complexed with their natural cytokine ligands (*i.e.* the “product complex”) (Fig. 1*B*). We used a soluble cytokine–receptor ECD complex to act as a “molecular cast” of the receptor dimer signaling conformation to isolate monovalent antibody fragments specific for the productive signaling complex, but not for the individual complex components. We hypothesized that the evolved antibodies would be specific for the “locked” receptor dimer stem region, binding to the receptor subunits in the geometry induced by the natural cytokine (Fig. 1*A*).

We focused our stapler engineering efforts on the interleukin-4 (IL-4) cytokine, a member of the family of common γ-chain (γ_c_) cytokines, which are critical for lymphocyte homeostasis and have been used to treat diseases such as cancer, chronic infection, and autoimmune disorders (5, 16). IL-4 signals through either a type I heterodimeric receptor comprised of IL-4 receptor-α (IL-4Rα) and the γ_c_ subunit or a type II heterodimeric receptor comprised of IL-4Rα and IL-13Rα1 (3, 34). The crystal structure of the type I IL-4 cytokine–receptor complex extracellular domains has been determined, revealing that the membrane-proximal domains of the receptor ECDs form a “stem region” of intimate receptor–receptor contact (Fig. S1) (34). We targeted these stem regions to isolate monovalent antibody fragments that bridge the type I IL-4 receptor dimer by simultaneously binding to the membrane-proximal domains of IL-4Rα and γ_c_. Crystallographic analysis showed that our evolved stapler indeed binds an interface spanning the two receptor subunits. We then extended our selection strategy to evolve scFvs that stabilize the interface between another γ_c_ cytokine, interleukin-2 (IL-2), and one of its receptor subunits. We showed that these scFvs enhance IL-2–receptor interaction and potentiate cytokine signaling in the context of an activity-impaired mutant. With this proof-of-concept demonstration for evolution of customized scFvs that recognize composite surfaces, our strategy could, in principle, enable discovery of stapler antibodies that modulate the activity of cytokines and other classes of natural ligands for a variety of therapeutic indications.

## Results

### Devising an evolutionary scheme for selection of dimerizing stapler scFvs

To screen for IL-4 staplers, we selected a diverse (10^9^ clones) yeast-displayed nonimmune human scFv library (35) against the type I IL-4 cytokine–receptor ternary complex (*i.e.* cytokine bound to two receptor subunits) through iterative rounds of magnetic-activated cell sorting (MACS) (Fig. 1*B*). There were two critical considerations for the selection of stapler scFvs: 1) the stability of the cytokine–receptor complex selection reagent that served as the “molecular cast” of the signaling dimer and 2) the use of a multistep selection scheme to positively select against these stabilized complexes and negatively select against the individual subunits to enrich for scFvs that specifically bind to receptor dimers.

To ensure stability of the target complex throughout the IL-4 stapler selections, we substituted the cytokine with an engineered variant of IL-4 (denoted “Super-4”) that has 3700-fold higher affinity for γ_c_ (36) and used the stabilized Super-4/IL-4Rα/γ_c_ ternary complex as our positive selection reagent. For each round of selection, we carried out two steps of negative selection against 1) γ_c_ and 2) IL-4/IL-4Rα binary complex preceding a positive selection step against the Super-4/IL-4Rα/γ_c_ target complex (Fig. S2). This sequence was iterated over six rounds of selection, decreasing only the concentration of the target complex to increase stringency.

### Staplers selectively and specifically bind the cytokine–receptor ternary signaling complex

Following selections, the library converged on three scFv clones denoted A2 (henceforth referred to as “IL-4 stapler”), A8, and A11 (Fig. S3). The yeast-displayed IL-4 stapler, A8, and A11 scFvs exhibited robust binding to the Super-4 ternary complex but not to any of the individual components or binary complex combinations thereof (Fig. 1*C* and Fig. S4). These scFvs were also specific for the Super-4/IL-4Rα/γ_c_ ternary complex and showed no reactivity with the IL-13/IL-4Rα/IL-13Rα1 or IL-2/IL-2Rβ/γ_c_ ternary complexes, indicating that receptor chain engagement occurred only in the context of the assembled IL-4Rα/γ_c_ heterodimer. On-yeast binding studies also revealed that IL-4 stapler bound weakly to the IL-4Rα chain and the Super-4/IL-4Rα binary complex, but binding was substantially enhanced in the presence of the full Super-4/IL-4Rα/γ_c_ ternary complex (Fig. 1*C*). Importantly, our engineered stapler scFvs did not bind to the type II IL-4 receptor complex composed of IL-4Rα/IL-13Rα1 and are thus highly specific for the IL-4Rα/γ_c_ type I IL-4 receptor heterodimer. This represents a major difference from endogenous IL-4, which cross-reacts with both type I and type II receptors and induces pleiotropic effects that have limited the utility of cytokine as a therapeutic to boost the type 2 helper T cell immune response (36).

The binding and specificity of recombinantly expressed IL-4 stapler scFv to the Super-4/IL-4Rα/γ_c_ target complex was evaluated via surface plasmon resonance. Consistent with on-yeast studies, IL-4 stapler scFv showed no binding to either Super-4 or γ_c_ and bound the IL-4Rα subunit with weak affinity, but it interacted strongly with the full ternary complex (Fig. 1*D*). The equilibrium binding constant (*K_D_*) for the stapler/ternary complex interaction was found to be 880 nm, whereas IL-4 stapler/IL-4Rα interaction was >10-fold weaker (Fig. 1*E* and Fig. S5), confirming the selectivity of IL-4 stapler for the active signaling complex. The A8 and A11 scFvs also bound the Super-4 ternary complex, but with affinities in the micromolar range (Fig. S4). By contrast, the IL-4 cytokine binds the IL-4Rα subunit alone with ∼150 pm affinity (37).

### Crystal structure of the ternary complex–bound IL-4 stapler reveals shared epitope between the IL-4Rα and γ_c_ subunits

**Figure 2.**
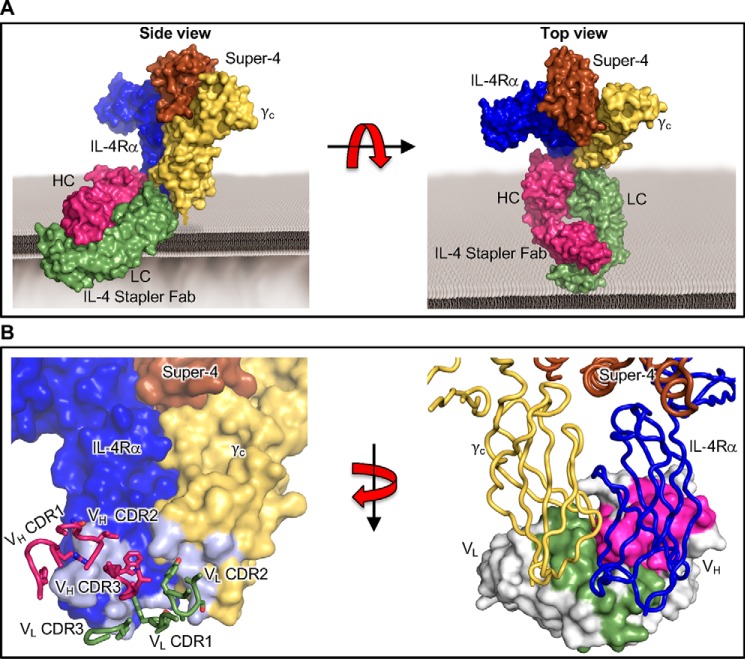
**Stapler recognizes a composite epitope between two receptor subunits to bridge the dimer interface.** *A*, *orthosteric views* of the crystallographic structure of the IL-4 stapler Fab fragment bound to the Super-4/IL-4Rα/γ_c_ ternary complex. *B*, *enlarged views* of the IL-4 stapler interfaces with the IL-4Rα and γ_c_ subunits. At the *left*, IL-4 stapler-binding sites on IL-4Rα and γ_c_ are depicted in *gray*, with interacting residues in the CDRs of the V_H_ (*magenta*) and V_L_ (*olive*) domains of the IL-4 stapler Fab shown as *sticks*. At the *right*, the CDRs on V_H_ (*magenta*) and V_L_ (*olive*) of the IL-4 stapler Fab are *shaded*.

To obtain structural evidence that the IL-4 stapler scFv recognizes a composite epitope formed by the conjunction of two receptor subunits (Fig. 1*A*), we reformatted the stapler scFv as a Fab fragment and determined the crystal structure of the Super-4/IL-4Rα/γ_c_ ternary complex bound to the stapler Fab to 3.1 Å resolution (Fig. 2*A*, Fig. S6, and Table 1). As selections and binding studies were performed in the presence of cytokine, we expected that the stapler would engage the membrane-proximal “stem regions” of the receptor subunits, distal from the site of cytokine engagement. The Fab heavy-chain (HC)/light-chain (LC) heterodimer axis coincides almost perfectly with the IL-4Rα/γ_c_ dimer interface. We also found close agreement between the orientation of the IL-4Rα and γ_c_ subunits in the presence of the stapler compared with the natural ternary complex. The receptor chains in the stapler-bound Super-4/IL-4Rα/γ_c_ ternary complex align closely with their conformations in the native IL-4 type I ternary complex (PDB code 3BPL, RMSD = 0.506 Å for IL-4Rα and RMSD = 0.643 Å for γ_c_). Furthermore, fixing the IL-4Rα1 subunit in both structures, the γ_c_ subunit is rotated by just 5.3° in the presence of the stapler. By comparison, a diabody that homodimerizes the erythropoietin receptor but elicits only weak agonist activity induces a 151° relative rotation in the receptor subunits (31). The IL-4 stapler Fab interface with the receptor subunits is partitioned between the variable domains; whereas the IL-4Rα interface is dominated by contacts with the variable heavy (V_H_) chain, the γ_c_ interface principally comprises contacts with the variable light (V_L_) chain (Fig. 2*B* and Fig. S7). For both the V_H_ and V_L_ domains, all three complementarity-determining regions (CDRs) are implicated in receptor interactions. The more extensive Fab interface with IL-4Rα compared with γ_c_ rationalizes the weak affinity observed between IL-4 stapler and IL-4Rα alone (Fig. 2*B* and Fig. S7).

**Table 1:** **Crystallographic statistics for Super-4/IL-4Rα/γ_c_/IL-4 stapler Fab complex** Shown are data collection and refinement statistics for solution of the crystal structure of IL-4 stapler Fab bound to the Super-4/IL-4Rα/γ_c_ ternary complex.

Parameters	Values
**Data collection**	
Wavelength	0.9999
Resolution range	63.1–3.10 (3.22–3.10)*^a^*
Space group	F 41 3 2
Unit cell	328.1, 328.1, 328.1, 90, 90, 90
Total reflections	493,043 (45,665)
Unique reflections	27,836 (1462)
Completeness (%)	95.0 (53.3)
Redundancy	17.7 (16.8)
Mean *I*/σ(*I*)	14.9 (0.92)
*R*-merge	0.275 (4.18)
CC1/2	0.997 (0.343)
**Refinement statistics**	
Reflections used in refinement	26,537 (1462)
Reflections used for *R*_free_	1327 (74)
*R*_work_	0.199 (0.316)
*R*_free_	0.256 (0.4118)
Number of non-hydrogen atoms	7209
Macromolecules	7091
Ligands	98
Solvent	20
Protein residues	922
RMSD (bonds)	0.003
RMSD (angles)	0.57
Ramachandran favored (outliers) (%)	95.0 (0)
Average *B*-factor	77.1
Macromolecules	76.6
Ligands	112
Solvent	77.1
Number of TLS groups	22

*^a^* Values in parentheses are for the highest-resolution shell.

### Isolating stapler scFvs that stabilize cytokine–receptor interactions

**Figure 3.**
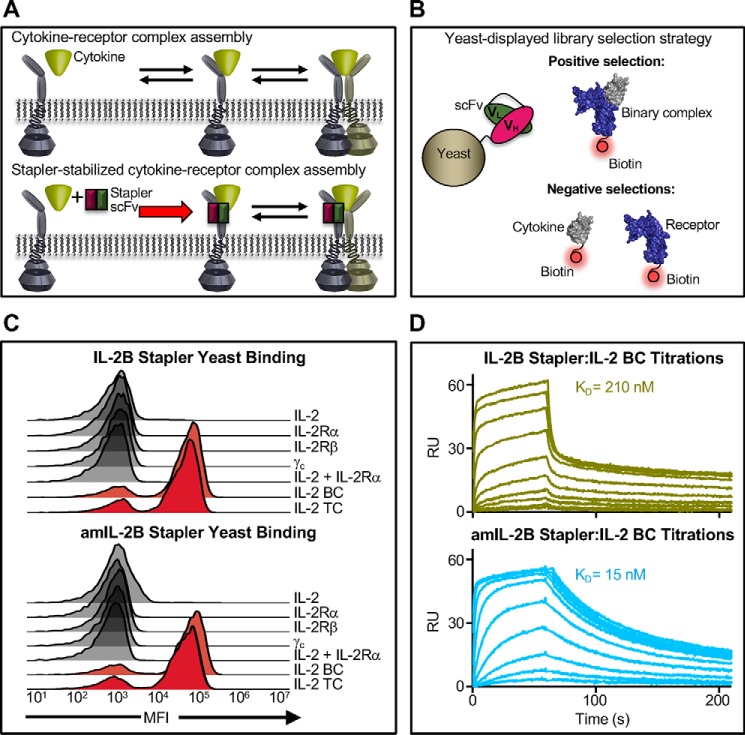
**Evolved staplers specifically engage cytokine–receptor binary complex with engineerable affinity.** *A*, *schematic* contrasting sequential assembly of a cytokine–receptor ternary complex in the absence (*top*) or presence (*bottom*) of a binary complex–stabilizing stapler scFv. *B*, yeast display platform diagram and selection strategy for evolution of cytokine–receptor binary complex stapler scFvs. *C*, binding of yeast-displayed IL-2B (*left*) or amIL-2B (*right*) stapler scFv to cytokine–receptor complexes and individual components thereof. Note the selective recognition of the IL-2/IL-2Rβ binary complex and compatibility with γ_c_ binding. *TC*, ternary complex. *D*, kinetic surface plasmon resonance binding profiles for the IL-2B stapler:IL-2 BC (*left*) and amIL-2B stapler:IL-2 BC (*right*) interactions. As shown in the *traces*, the affinity-matured stapler variant improves affinity 14-fold through deceleration of the dissociation rate. *K_D_* values were calculated by fitting equilibrium titration curves to a logistic model via nonlinear regression.

Given our success in engineering scFvs that recognize epitopes that span multiple cytokine receptor subunits, we attempted to extend the stapling concept to select for interface-bridging scFvs that stabilize interactions between cytokines and their cognate receptor subunits (Fig. 3*A*). In particular, we aimed to evolve a single-chain species that potentiates the interaction between the interleukin-2 (IL-2) cytokine and one of its receptor subunits. IL-2 signals through either a high-affinity (*K_D_* ≈ 10 pm) heterotrimeric receptor comprised of the IL-2Rα, IL-2Rβ, and γ_c_ chains or an intermediate-affinity (*K_D_* ≈ 1 nm) heterodimeric receptor consisting of only the IL-2Rβ and γ_c_ chains (Fig. S8) (38–40). The IL-2Rα chain is not implicated in signaling but rather calibrates IL-2 sensitivity through its differential expression levels on various cell types. IL-2Rα is highly expressed on regulatory T (T_Reg_) cells but virtually absent from naive CD8^+^ T and natural killer (NK) cells; hence, TReg cells are 100-fold more responsive to IL-2 than these immune effector cell types (38, 41). A variant of IL-2 with 200-fold enhanced affinity for the IL-2Rβ chain (denoted “Super-2”) was previously engineered to sensitize IL-2Rα–deficient cells to IL-2. Super-2 promoted expansion of cytotoxic T cells to effectively inhibit tumor growth in mice (42). We hypothesized that an scFv stapler that stabilized the IL-2/IL-2Rβ interaction would recapitulate the behavior of Super-2. Moreover, therapeutic use of an exogenous antibody offers several advantages over administration of a cytokine mutant, such as improved serum half-life (23, 24), controllable dose-dependent activation of IL-2–responsive cells, and the ability to selectively modulate rather than compete with endogenous IL-2.

Recently, Kuruganti *et al.* (32) selected antibody fragments that stabilize the IFN/interferon receptor 2 (IFNAR2) binary complex to potentiate IFN signaling. Following a selection scheme similar to those used for IL-4 stapler discovery (Fig. 3*B* and Fig. S9), we evolved an scFv that uniquely recognizes the IL-2/IL-2Rβ binary complex but not either individual component thereof. We performed iterative rounds of selection against the aforementioned scFv library (35), clearing independently against 1) Super-2 and 2) IL-2Rβ prior to positively selecting for binders to the Super-2/IL-2Rβ complex, again capitalizing on the enhanced affinity of a mutant cytokine (Super-2) to stabilize the target complex (Fig. 3*B*). Selections converged on a single scFv clone (denoted the IL-2 binary complex stapler, or “IL-2B stapler”) that was then affinity-matured through selection of a site-specific mutagenic library of the V_H_ and V_L_ CDR3 regions (Fig. S10).

**Figure 4.**
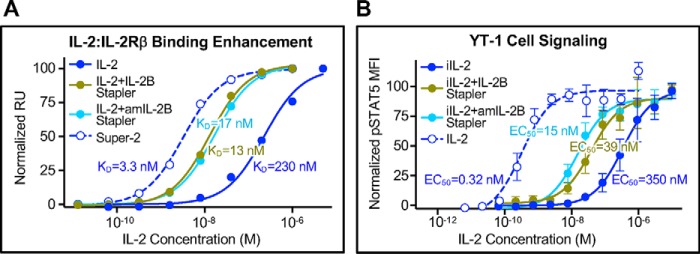
**Binary complex staplers strengthen cytokine–receptor interactions to potentiate signaling of an affinity-impaired cytokine variant.** *A*, equilibrium surface plasmon resonance titrations of the IL-2:IL-2Rβ binary complex interaction without scFv present (*filled blue circles*) and with IL-2BC stapler scFv (*gold circles*) or amIL-2B stapler scFv (*cyan circles*) compared with the Super-2:IL-2Rβ interaction (*unfilled blue circles*). *K_D_* values were calculated by fitting equilibrium titration curves to a logistic model via nonlinear regression. IL-2B staplers increase the IL-2:IL-2Rβ interaction affinity ∼15-fold. *B*, YT-1 cell STAT5 phosphorylation responses to impaired affinity IL-2 (*iIL-2*) without scFv present (*filled blue circles*) and with saturating levels of IL-2B stapler (*gold circles*) or amIL-2B stapler (*cyan circles*) present compared with WT IL-2 (*unfilled blue circles*). EC_50_ values are indicated. *Error bars*, S.D.

Both the parent IL-2B stapler and the affinity-matured variant thereof (denoted the “amIL-2B stapler”) bound the IL-2/IL-2Rβ binary complex, but not the cytokine or receptor independently (Fig. 3*C*). IL-2B staplers also bound the IL-2/IL-2Rβ/γ_c_ ternary complex, indicating that they recognize an epitope that is noncompetitive with γ_c_ engagement of the IL-2/IL-2Rβ binary complex, which is critical for signaling activity. The IL-2B stapler bound the stabilized Super-2/IL-2Rβ binary complex with similar affinity (*K_D_* = 210 nm) to the IL-2/IL-2Rβ interaction (42), and the amIL-2BC stapler exhibited a 14-fold higher affinity for the binary complex (*K_D_* = 15 nm; Fig. 3*D* and Fig. S11*A*). Both IL-2B stapler scFvs enhanced the IL-2/IL-2Rβ interaction ∼15-fold, although this improvement was less dramatic than the 70-fold enhancement observed with the Super-2 cytokine mutant (Fig. 4*A*). Due to this more modest affinity improvement and the high potency of WT IL-2 signaling, IL-2B staplers did not potentiate cytokine activity on IL-2–responsive cells, as measured by phosphorylation of the downstream transcription factor signal transducer and activator of transcription 5 (STAT5, Fig. S11*B*). We speculated that the effect of the staplers would be more pronounced in the context of an impaired IL-2 variant reported by Shanafelt *et al.* (43), which exhibits weakened activity due to its reduced affinity for IL-2Rβ. This variant (which we denote “iIL-2”) has been shown to selectively bias cytokine activity toward particular immune cell subsets based on their IL-2 receptor expression profiles (43). IL-2B stapler and amIL-2B stapler enhanced iIL-2–induced STAT5 activity 9- and 23-fold, respectively (Fig. 4*B*), establishing the potential utility of binary complex stapler scFvs to selectively potentiate immune cells for such applications as T cell activation in cancer immunotherapy (38, 44). Taken together, our biophysical, structural, and functional characterization of staplers demonstrates that through stringent selection against stabilized cytokine–receptor complexes, we have engineered antibody fragments that specifically recognize and modulate the stability of protein interfaces.

## Discussion

The ability to isolate scFvs that use a single binding site to bridge bimolecular interactions from a naive antibody fragment library presents an exciting opportunity in antibody engineering. A naive scFv library of diversity 10^9^, which was derived from pooled spleen and lymph nodes of 58 human donors (35), yielded three unique IL-4 ternary complex stapler clones and one unique IL-2 binary complex stapler clone, and it will be interesting to explore the frequency of stapler antibodies in both natural and synthetic libraries going forward. Moreover, our IL-2 cytokine–receptor stapler demonstrates the unexpected ability to stabilize a protein interface. Previous work has shown that catalytic antibodies can be elicited by raising antibodies that target the transition state of reactions (45, 46). Our biophysical and functional data suggest that staplers instead act through stabilization of the receptor heterodimer to bias the equilibrium toward the dimeric over the monomeric receptor state.

Whereas our study is limited to a proof of concept and *in vitro* studies, a potential advantage of the stapler platform is that it allows for modulation of the heterodimerization properties of membrane-bound receptors through recognition of specific receptor complexes. Regulating heterodimer distribution on cells has value from both a research discovery and a therapeutic perspective (36). Altered dimerization patterns could have profound functional implications, particularly for cytokine systems such as IL-4, which signal through formation of either a type I or type II receptor complex to evoke distinct immune behavior (3, 34). In addition, the specificity of staplers makes a compelling case for their use in targeted activation of transplanted cells. Previous efforts have been employed to engineer natural cytokines (22, 36, 42, 47), to design synthetic cytokine fusions (48), or to computationally engineer *de novo* cytokine mimetics (49) to redirect cytokine activities to specific cell subsets. The stapler strategy allows for an additional layer of control through administration of a molecule that is noncompetitive with natural cytokine activity.

The engineering opportunities available to the scFv scaffold enable possibilities for molecular therapeutic design. Stapler scFvs can be conjugated to scFvs that engage cell surface markers to form bispecific antibodies that selectively modulate dimerization behavior on particular immune cell subsets for targeted disease therapy. Efforts to achieve cell subset-specific signaling by directly fusing cytokines to surface marker–directed antibodies have been unsuccessful, as the high potency of cytokines nullifies the effect of targeting antibodies, resulting in nonspecific activation of many immune cell subtypes (50–52). In contrast, the affinities of the stapler scFv and cell surface marker–targeted scFvs can be easily evolved through manipulation of the CDR loop regions (as was done in our binary stapler affinity maturation) to finely control their relative potencies and thereby optimize functional selectivity. Furthermore, discovery and structural characterization of the IL-4 stapler paves the way for engineering staplers that modulate other γ_c_-engaging cytokines (including IL-7, IL-9, and IL-21) (16). In summary, we have established a new evolutionary approach to isolate a novel class of stapler scFvs. These engineered molecules offer insight into the biophysical requirements for dimerization and signal activation, and they provide a structural framework for the development of targeted immunotherapeutics.

## Experimental procedures

### Protein expression and purification

Human IL-2 and variants thereof (amino acids 1–133), the IL-2Rβ ectodomain (amino acids 1–214), γ_c_ (amino acids 34–232), IL-2Rα (amino acids 1–217), IL-15 (amino acids 1–114), and IL-15Rα (amino acids 1–67) were expressed and purified using a baculovirus expression system as described (39). Super-2 refers to the previously reported IL-2 variant denoted clone H9 (42). Additionally, human IL-4 and variants thereof (amino acids 1–129), IL-13 (amino acids 1–112), the IL-4Rα ectodomain (amino acids 1–202), and the IL-13Rα1 ectodomain (amino acids 1–310) were expressed and purified using a baculovirus expression system (34). The sequence for the Super-4 variant of IL-4 was provided previously (36). scFv clones isolated from selections were expressed and purified in the baculovirus system via transfer of their variable regions into the pAcGP67A vector (BD Biosciences) with an N-terminal gp67 signal peptide and a C-terminal hexahistidine tag. scFvs were expressed with the V_H_ and V_L_ chains separated by a 15-amino acid (Gly_4_Ser)_3_ linker. To prevent isomerization, scFvs were eluted in 10 mm HEPES, pH 7.3, 200 mm imidazole in the presence of 15% glycerol and 500 mm NaCl and then exchanged into standard HEPES-buffered saline (10 mm HEPES, pH 7.3, 150 mm NaCl). The V_H_ and V_L_ chains of stapler scFvs were delineated using the International Immunogenetics Information System (IMGT) V-QUEST tool (53, 54). For IL-4 stapler Fab expression, the identified V_H_ followed by the human IgG1 constant heavy 1 (CH1) domain and hinge region and the identified V_L_ domain followed by the human κ constant light (CL) domain were separately cloned into the pAcGP67A vector containing 3C protease-cleavable basic and acidic leucine zippers, respectively, for high-fidelity Fab chain pairing (55, 56). The resulting Fab HC and LC constructs were transfected independently, and their corresponding viruses were co-titrated to determine optimal infection ratios for equivalent expression of the two chains. The secreted IL-4 stapler Fab was treated with 3C protease to remove the leucine zippers prior to size-exclusion chromatographic purification.

For biotinylated protein expression, Super-4, IL-4Rα, IL-13Rα1, or γ_c_ with a C-terminal biotin acceptor peptide, LNDIFEAQKIEWHE, were co-expressed with the BirA ligase enzyme in the presence of excess biotin (100 mm), or the purified proteins were biotinylated *in vitro* using the soluble BirA ligase enzyme in 0.5 mm Bicine, pH 8.3, 100 mm ATP, 100 mm magnesium acetate, and 500 mm biotin (Sigma). For surface plasmon resonance (SPR) studies, purified stapler scFvs were biotinylated using the EZ-Link Sulfo-NHS-LC-biotinylation kit (Pierce) according to the manufacturer's protocol. All proteins contained C-terminal hexahistidine tags and were isolated by nickel chromatography. They were then further purified to >98% homogeneity by size-exclusion chromatography on a Superdex 200 column (GE Healthcare), equilibrated in 10 mm HEPES (pH 7.3) and 150 mm NaCl.

### Tissue culture

YT-1 human NK cells (57) were cultured in RPMI complete medium (RPMI 1640 medium supplemented with 10% fetal bovine serum, 2 mm l-glutamine, minimum nonessential amino acids, sodium pyruvate, 25 mm HEPES, and penicillin-streptomycin (Gibco)). The Ramos human B lymphocyte cell line (58) was grown in RPMI containing 10% FBS, l-glutamine (2 mm), and penicillin/streptomycin (Gibco). Prior to stimulation, Ramos cells were cultured overnight in modified growth medium containing only 2% FBS (“starved”).

### Yeast library selection of stapler scFvs

General yeast display methodologies were modified from protocols described previously (42, 59, 60). The previously described human nonimmune scFv yeast library (35) was kindly provided by K. Dane Wittrup and was used to isolate the IL-4 and IL-2 stapler scFvs. For all selection schemes, the first round of sorting was performed with 1 × 10^10^ cells from the naive yeast library to achieve 10-fold coverage of the number of transformants, and subsequent rounds used 1 × 10^8^ yeast cells. Tetramers were formed by incubating a 4:1 ratio of biotinylated protein to Alexa 647–conjugated streptavidin (SA-647) for 15 min on ice. Magnetic bead selections were performed using LS MACS separation columns (Miltenyi Biotec) according to the manufacturer's protocol. The naive scFv library and each iterative panel of selected clones were grown fresh overnight at 30 °C in SDCAA liquid medium (pH 4.5), followed by induction in SGCAA liquid medium (pH 4.5) for 2 days at 20 °C.

### IL-4 ternary complex stapler scFv selections

In the first round of selection for the IL-4 ternary complex staplers, yeast-displayed scFvs were incubated with biotinylated γ_c_ bound to magnetic beads coated with SA (400 nm γ_c_ in 250 μl of SA-coated beads) (Miltenyi Biotec) in the presence of 1 μm nonbiotinylated IL-4Rα and Super-4, an IL-4 mutant that exhibits 3700-fold higher affinity for γ_c_ than WT IL-4 (36), in PBE solution (PBS, pH 7.2, 1% BSA, and 1 mm EDTA) for 2 h at 4 °C. In each subsequent round of selection, two steps of negative selection and one step of positive selection were carried out in sequence. In a first step, yeast were stained with biotinylated γ_c_ that had been tetramerized via incubation with SA-647 (500 nm) for 1 h in PBE at 4 °C and negatively selected by incubating the yeast with 50 μl of anti-Alexa 647 microbeads (Miltenyi Biotec) in PBE for 15 min at 4 °C. In a second step, the flow-through yeast from the first step were incubated with SA-647–tetramerized biotinylated IL-4Rα (500 nm) and 1 μm of nonbiotinylated Super-4 for 1 h in PBE at 4 °C. The yeast were then negatively selected via incubation with 50 μl of anti-Alexa 647 microbeads (Miltenyi Biotec) in PBE for 15 min at 4 °C. In a final step, the flow-through yeast from the second step were incubated with SA-647–tetramerized biotinylated γ_c_ (500 nm) in the presence of 1 μm nonbiotinylated IL-4Rα and Super-4 for 1 h in PBE at 4 °C and positively selected by incubation with 50 μl of anti-Alexa 647 microbeads (Miltenyi Biotec) in PBE for 15 min at 4 °C. This workflow was repeated over six rounds of selection in which only the concentration of the positive selection reagents was decreased to isolate scFvs specifically binding the IL-4 ternary complex with high affinity. Three scFv clones, denoted A2 (the “IL-4 stapler”), A8, and A11, were isolated, and all exhibited specific binding for the IL-4 ternary complex and no binding for the individual components of that complex (*i.e.* Super-4, IL-4Rα, or γ_c_). The complementarity-determining region 3 (CDR3) domain sequences of isolated IL-4 staplers are provided in Fig. S3.

### IL-2 binary complex stapler scFv selections and affinity maturation

In the first round of selection for an IL-2 binary complex stapler, the naive library of yeast-displayed scFvs was stained with biotinylated IL-2Rβ bound to SA-coated magnetic beads (400 nm IL-2Rβ in 250 μl of SA-coated beads) (Miltenyi Biotec) in the presence of 1 μm nonbiotinylated Super-2, a variant of IL-2 with 200-fold greater affinity for the IL-2Rβ receptor subunit (42), for 2 h at 4 °C. In each subsequent round of selection, two negative selection steps and one positive selection step were conducted sequentially. In a first step, yeast were stained with SA-647–tetramerized biotinylated Super-2 (500 nm) for 1 h in PBE at 4 °C. Negative selections were performed by incubating the yeast with 50 μl of anti-Alexa 647 microbeads (Miltenyi Biotec) in PBE for 15 min at 4 °C. In a second step, the flow-through yeast from the first step were incubated with SA-647–tetramerized biotinylated IL-2Rβ (500 nm) for 1 h in PBE at 4 °C. Negative selections were again performed by incubating the yeast with 50 μl of anti-Alexa 647 microbeads (Miltenyi Biotec) in PBE for 15 min at 4 °C. In a final step, the flow-through yeast from the second step were incubated with SA-647–tetramerized biotinylated IL-2Rβ (500 nm) and 1 μm nonbiotinylated Super-2 for 1 h at 4 °C. Positive selections were conducted by incubating the yeast with 50 μl of anti-Alexa 647 microbeads (Miltenyi Biotec) in PBE for 15 min at 4 °C. Iterations of this workflow were repeated over six rounds of selection in which only the concentrations of the positive selection reagents were decreased to isolate scFv clones specifically binding with high affinity to the IL-2 binary complex. Selections converged on a single clone, the IL-2 binary complex stapler (“IL-2B stapler”).

The site-directed mutagenic library used for affinity maturation of IL-2B stapler was constructed via assembly PCR of 16 primers spanning the V_H_ and V_L_ chains of the scFv. The following degenerate codons were used: Ala-104, RYK; Val-105, RYK; Ser-106, DMY; Gly-107, RBY; Thr-108, DMY; Phe-109, HWY; Tyr-236, WWY; Asp-237, VAK; Asp-238, VAK; Asn-239, VAK; Tyr-240, WWY. The assembly PCR was carried out using Pfu Ultra DNA polymerase (Stratagene), and the product was further amplified using primers containing sequence homology to pCT302 for recombination inside the yeast. Insert DNA was combined with linearized vector backbone and electroporated into EBY100 yeast as described previously. Electroporation yielded a library of 1 × 10^8^ transformants. Selections were performed as detailed for the IL-2 binary complex stapler, but with 10-fold reduced concentrations of positive selection reagents to heighten stringency. Selections converged on a single clone, the affinity-matured IL-2 binary complex stapler (amIL-2B stapler). The CDR3 domain sequences of the IL-2 binary complex stapler (IL-2B stapler) and the affinity-matured IL-2 binary stapler (amIL-2B stapler) are provided in Fig. S9.

### Yeast surface stapler scFv-binding studies

Yeast displaying stapler scFvs (2 × 10^5^/well) were plated in a 96-well plate and incubated in PBE buffer containing a 1 μm concentration of the indicated biotinylated IL-4 or IL-2 complex components for 2 h at room temperature. Cells were then washed and incubated for 20 min at 4 °C with 50 nm SA-647 diluted in PBE. After a final wash, cells were analyzed for binding using an Accuri C6 flow cytometer (BD Biosciences). Representative plots from one of three independent experiments are presented. Cytometry data were analyzed using FlowJo software (Tree Star, Inc.).

### Surface plasmon resonance binding measurements

Binding interactions were characterized via SPR studies using Biacore SA sensor chips (GE Healthcare) on a Biacore T100 instrument at 25 °C. All recombinantly produced proteins were purified via size-exclusion chromatography on an FPLC instrument and filtered through a 0.22-μm centrifugal column prior to SPR analysis. Biotinylated scFv stapler clones were immobilized to the chip surface at low density (RU_max_ < 200 response units), and measurements were performed at a flow rate of 30 ml/min to minimize mass transport contributions and prevent rebinding of the analyte. Dilutions of the analytes (IL-4 cytokine–receptor complex components) were exposed to the surface channels for 60 s, and dissociation was measured for 180–300 s. For the case of protein complexes, complex components were incubated for 30 min at room temperature prior to SPR analysis. An irrelevant scFv was used in the reference channel to subtract nonspecific binding from the measurements. Experiments were performed in HBS-P+ buffer (GE Healthcare) supplemented with 0.2% BSA. Data were visualized and processed using the Biacore T100 evaluation software (GE Healthcare). Equilibrium titration curves were fitted to a logistic model via nonlinear regression, and equilibrium dissociation constant (*K_D_*) values were determined using GraphPad Prism analysis software assuming 1:1 binding interactions.

### Crystallization and data collection

For crystallographic analysis, the Super-4, IL-4Rα, and γ_c_ viruses were co-expressed in insect cells in the presence of 5 μm kifunensine, added to culture at the time of infection. The resulting kifunensine-sensitized Super-4 ternary complex was purified via nickel chromatography and then treated overnight at 4 °C with 1:100 (w/w) endoglycosidases F1 and H to remove *N*-linked glycans and with 1:100 (w/w) carboxypeptidases A and B (Sigma) to remove C-terminal hexahistidine tags. The complex was then purified by size-exclusion chromatography. Separately, the IL-4 stapler Fab was secreted in insect cells via co-infection of heavy- and light-chain viruses and purified via nickel chromatography. The assembled Fab was then treated overnight at 4 °C with 1:1000 (w/w) 3C protease to remove acidic and basic leucine zippers and hexahistidine tags and purified by size-exclusion chromatography. Stoichiometrically equivalent amounts of the purified Super-4/IL-4Rα/γ_c_ ternary complex and the IL-4 stapler Fab were co-incubated for 4 h at 4 °C to allow for complex formation, and the resulting Fab-bound cytokine–receptor complex was purified by co-elution over a Superdex-200 size-exclusion chromatography column and concentrated to >10 mg/ml. Super-4/IL-4Rα/γ_c_/IL-4 stapler Fab complex crystals were grown in sitting drops at 22 °C in 22.5% PurePEG (Microlytic), 0.1 m sodium citrate, pH 5.5, and 0.6 m ammonium nitrate. Crystals were cryoprotected with the addition of 30% ethylene glycol (Sigma) and flash-cooled in liquid nitrogen. A 3.1 Å data set was collected at beamline 8.2.1 at the Advanced Light Source. Diffraction data were processed using HKL2000. Crystallographic data collection and refinement statistics are reported in Table 1.

### Structure determination and refinement

The Super-4/IL-4Rα/γ_c_/IL-4 stapler Fab complex structure was solved by molecular replacement using PHASER (61). The IL-4 ternary complex structure reported previously (PDB code 3BPL) (34) was used to model Super-4, IL-4Rα, and γ_c_, and models of the IL-4 stapler Fab heavy and light chains were obtained from the previously solved structure of a human Fab (PDB code 3SOB) (62). Iterative model rebuilding and refinement were performed using the Phenix software (63) and COOT (Crystallographic Object-Oriented Toolkit) (64). For initial refinement, rigid body, coordinate, and real-space refinement were used with individual atomic displacement parameter refinement. Translation, libration, and screw-rotation refinement was added in later iterations. Ramachandran and rotamer analysis were performed using MolProbity (65). Atomic interactions were identified using the PISA (protein interfaces, surfaces, and assemblies) service at the European Bioinformatics Institute (66). Structural figures were created using PyMOL (The PyMOL Molecular Graphics System, version 2.0 Schrödinger, LLC). Crystallographic software was installed and configured by SBGrid (67).

### YT-1 signal response experiments and phospho-flow cytometric analysis

Approximately 3 × 10^5^ YT-1 cells were plated in each well of a 96-well plate, washed with FACS buffer, and resuspended in FACS buffer containing serial dilutions of stapling scFvs or cytokines (used as positive controls for signal activation). Cells were stimulated for 20 min at 37 °C and immediately fixed by the addition of formaldehyde to 1.5% followed by incubation for 10 min at room temperature. Cells were then permeabilized with 100% ice-cold methanol for 30 min at 4 °C. The fixed and permeabilized cells were washed twice with FACS buffer and incubated with Alexa 488–conjugated anti-STAT5 pTyr-694 antibody (BD Biosciences) diluted in FACS buffer for 2 h at room temperature. Cells were then washed twice in FACS buffer, and mean fluorescence intensity was quantified on an Accuri C6 flow cytometer. Dose-response curves were fitted to a logistic model, and half-maximal effective concentrations (EC_50_ values) were computed in the GraphPad Prism data analysis software after subtraction of the mean fluorescence intensity of unstimulated cells and normalization to the maximum signal intensity induced by cytokine stimulation.

## Author contributions

J. B. S. and I. M. evolved, biophysically characterized, and functionally validated stapler antibody fragments; J. B. S., I. M., and K. M. J. performed crystallographic studies of the stapler/cytokine receptor complexes and determined and refined these structures; J. B. S., I. M., K. M. J., and C. S. S. designed and prepared the cytokine proteins and recombinant antibody fragments used in this work; J. B. S., I. M., K. M. J., and K. C. G. designed experiments; J. B. S. and I. M. prepared the figures for this manuscript; J. B. S., I. M., and K. C. G. wrote the paper; and K.C.G. conceived of, designed, and supervised the research.

## Supplementary Material

Supporting Information

## Footnotes

4 The abbreviations used are:

ECD: extracellular domain
JAK: Janus kinase
STAT: signal transducer and activator of transcription
scFv: single-chain variable fragment
IL: interleukin
γ_c_: γ-chain
MACS: magnetic-activated cell sorting
HC: heavy-chain
LC: light-chain
PDB: Protein Data Bank
RMSD: root mean square deviation
V_H_: variable heavy
V_L_: variable light
CDR: complementarity-determining region
T_Reg_: regulatory T
NK: natural killer
SPR: surface plasmon resonance
SA: streptavidin.
