# Supplementary material for: A strategy for the selection of monovalent antibodies that span protein dimer interfaces
Source: J Biol Chem. 2019 Aug 6;294(38):13876–86. doi: 10.1074/jbc.RA119.009213 (PMC6755802; doi:10.1074/jbc.RA119.009213)
Supplement: Supporting Information [file supp_294_38_13876__index.html]

A strategy for the selection of monovalent antibodies that span protein dimer interfaces — Selection of antibodies that span protein dimer interfaces — A strategy for the selection of monovalent antibodies that span protein dimer interfaces — EDITORS' PICK: Antibodies that span protein dimer interfaces — Supporting Information 

# A strategy for the selection of monovalent antibodies that span protein dimer interfaces

## Supporting Information

- Supporting Information (to be published online) - Supporting Information
