# Supplementary material for: A strategy for the selection of monovalent antibodies that span protein dimer interfaces
Source: J Biol Chem. 2019 Aug 6;294(38):13876–86. doi: 10.1074/jbc.RA119.009213 (PMC6755802; doi:10.1074/jbc.RA119.009213)
Supplement: Supporting Information [file supp_RA119.009213_152636_1_supp_363588_ptptwt.pdf]

Figure S1

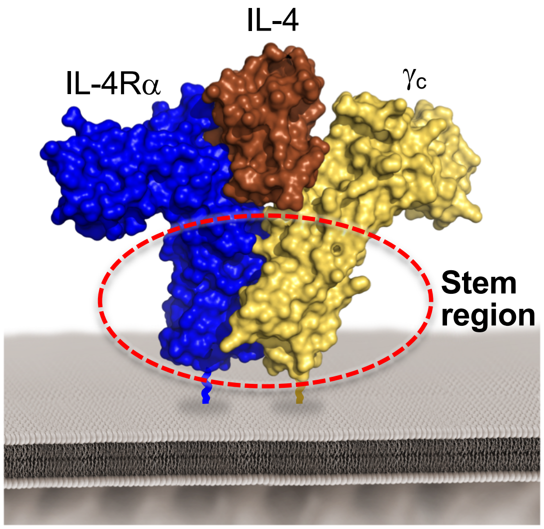

IL-4 cytokine-receptor ternary complex structure.
Crystal structure of the type I IL-4 (brown)/IL-4Rα (blue)/γ_c (yellow) ternary complex (PDB ID 3BPL). The complementary interface between the membrane-proximal D2 domains of the two receptor subunits ('stem region') is circled in red.

Figure S2

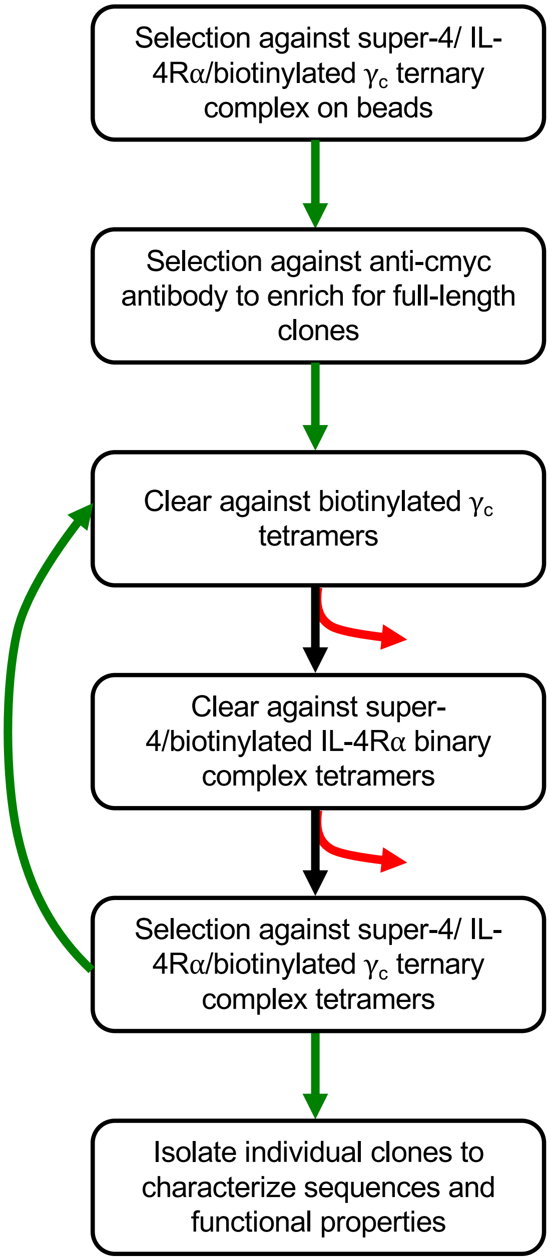

IL-4 ternary complex stapler selection strategy.

Flow diagram detailing the selection scheme used to to isolate stapler scFvs from a naïve human nonimmune yeast-displayed scFv library. Note the use of super-4 in place of the IL-4 cytokine to stabilize the target complex.

Figure S3

	V_H CDR3	V_L CDR3
IL-4 Stapler	C A R F S G W L G T G - D C W	C Q Q S Y S T P W T F
scFv A8	C A T G T W I Q Y Y G L D V W	C Q Q Y G T S P H T F
scFv A11	C A R S S Y S R G W S V D V W	C Q Q R S N W I - T F

IL-4 stapler variable domain sequences.

Sequences of the variable heavy (V_H) and variable light (V_L) chain third complementarity determining regions (CDR3s) for the three isolated IL-4 stapler scFvs.

Figure S4

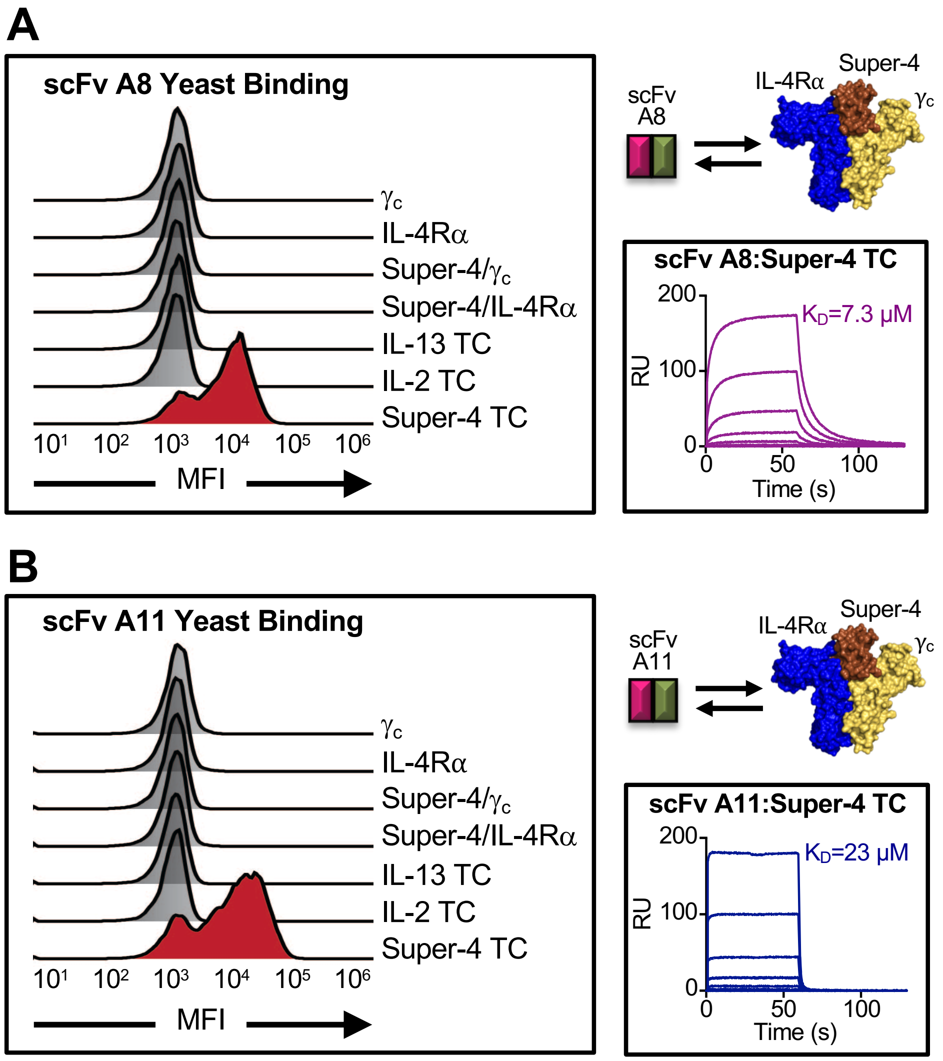

Evolved stapler scFvs selectively bind the active IL-4 cytokine-receptor ternary complex. Binding of yeast-displayed scFv A8 (**A**) and scFv A11 (**B**) staplers to soluble cytokine-receptor complexes or components thereof. Both stapler scFvs specifically recognize the fully assembled super-4/IL-4Rα/γ_c TC. Surface plasmon resonance kinetic binding profiles detailing the interactions between scFv A8 or scFv A11 and the super-4/IL-4Rα/γ_c TC are shown at *right*. K_D values values were calculated by fitting equilibrium titration curves to a logistic model via non-linear regression. The top curve represents a concentration of 20 μM and subsequent curves represent threefold serial dilutions.

Figure S5

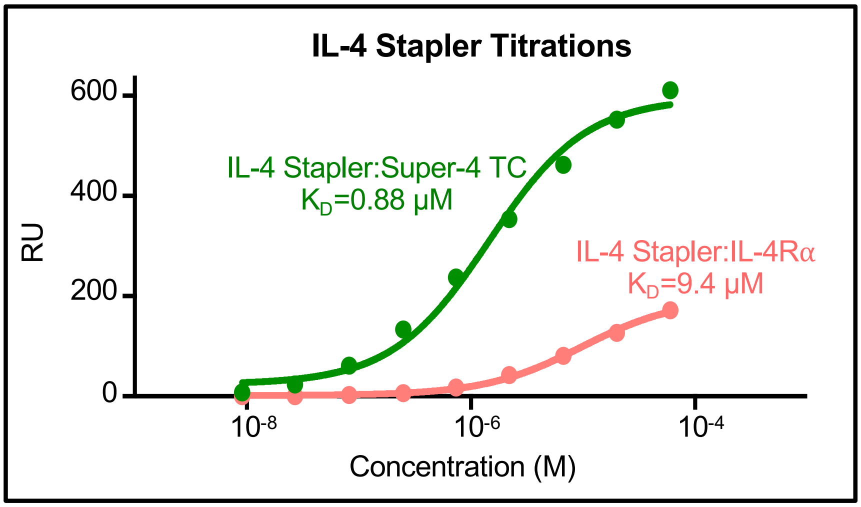

IL-4 stapler engages the free IL-4Rα subunit with low affinity.
Comparison of the equilibrium surface plasmon resonance titrations of IL-4 stapler scFv binding to the super-4 TC versus the IL-4Rα subunit only. K_D values were calculated by fitting equilibrium titration curves to a logistic model via non-linear regression.

Figure S6

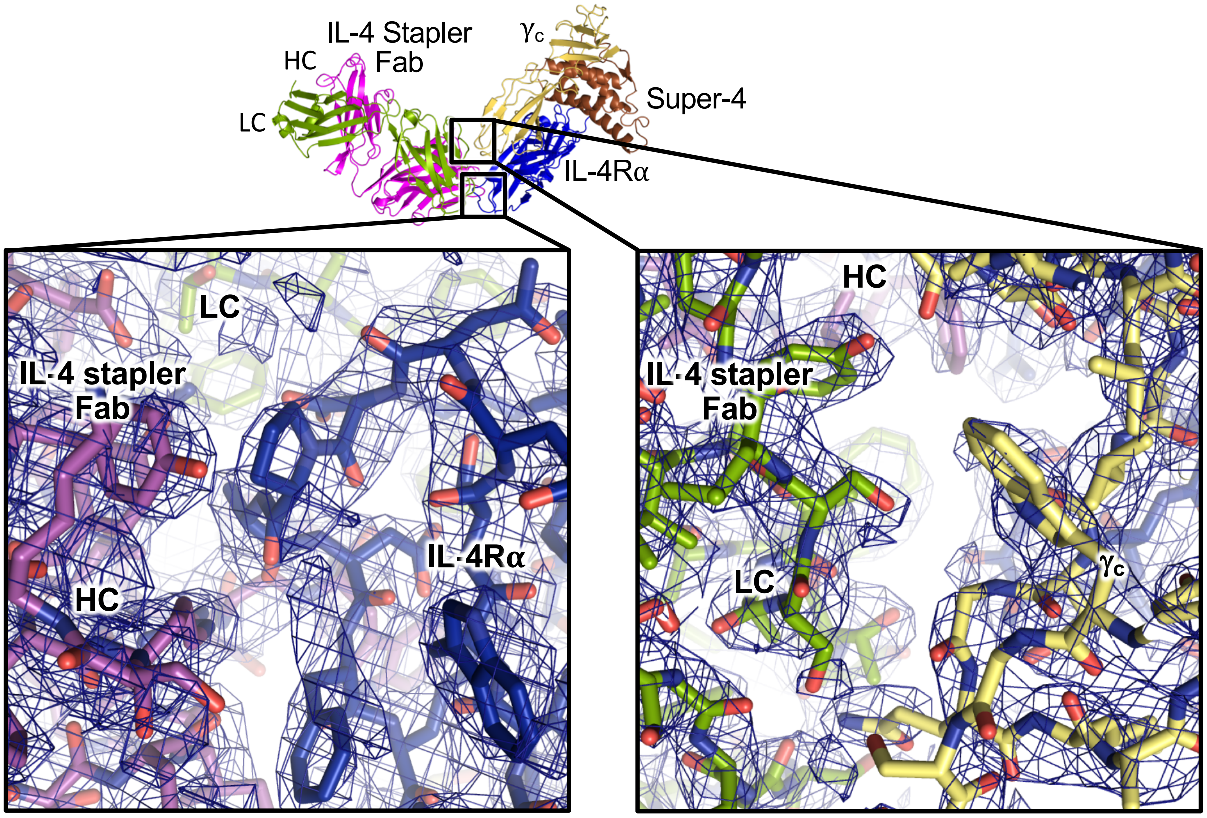

Stapler heavy and light chains exhibit partitioned engagement of the IL-4Rα and γ_c receptor subunits.

Crystal structure (3.1 Å resolution) of the full IL-4 Stapler Fab (HC shown in magenta and LC shown in olive) bound to the super-4 (brown)/IL-4Rα (blue)/γ_c (yellow) ternary complex. Details of the IL-4 Stapler light chain/γ_c heavy chain/IL-4Rα (*left*) and the IL-4 Stapler light chain/γ_c (*right*) interfaces are shown overlaid with a composite, simulated-annealing omit 2mFo - DFc map contoured at 1 sigma (navy).

Figure S7

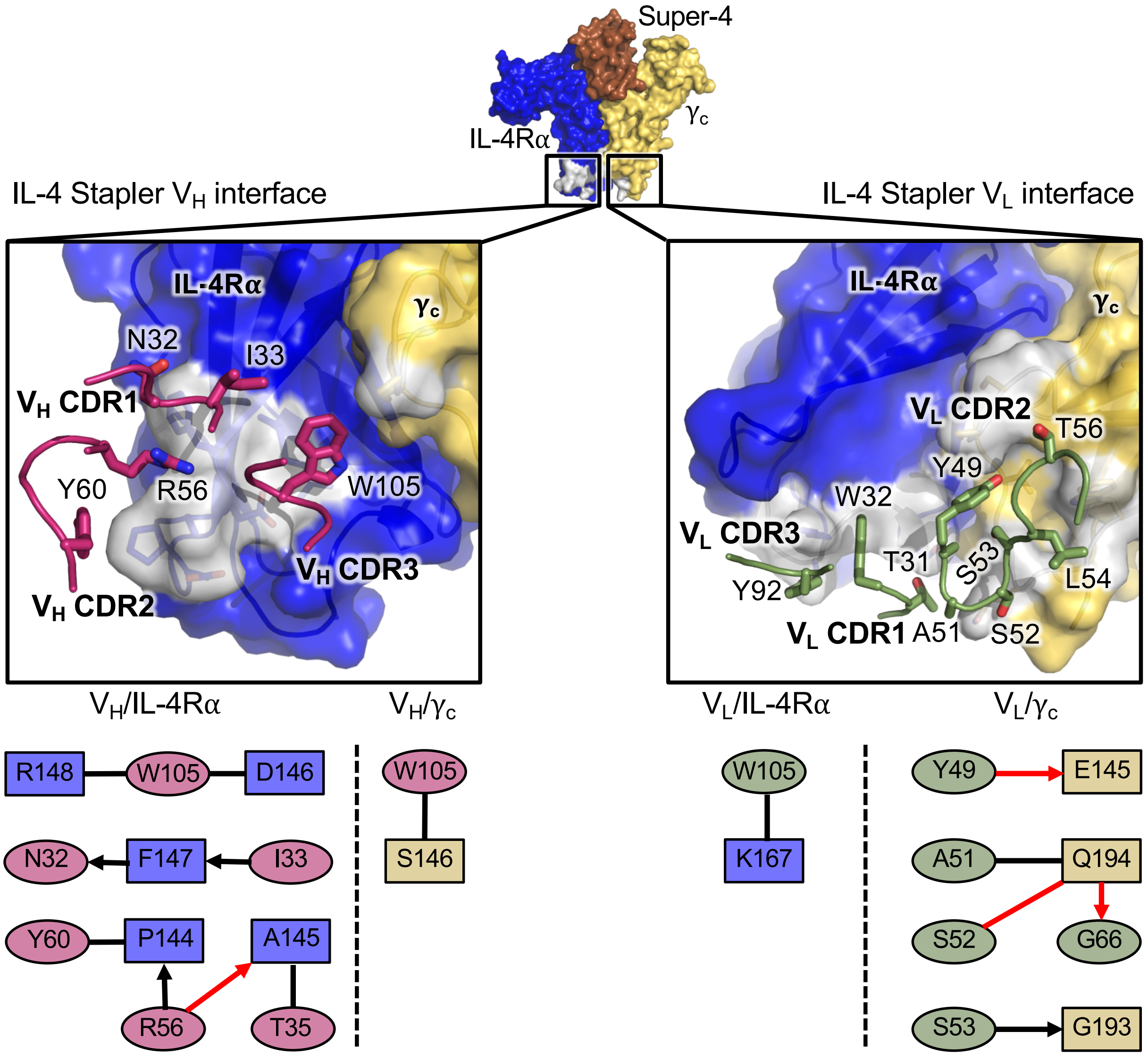

Stapler binds IL-4Rα/γ_c heterodimer through contacts in all three complementarity determining regions (CDRs) of both heavy and light chains.

A surface representation of the super-4 (brown)/IL-4Rα (blue)/γ_c (yellow) ternary complex with IL-4 Stapler Fab epitopes colored in gray is shown at *top*. Enlarged views of the interfaces between IL-4 Stapler V_H (magenta, *left*) and V_L (olive, *right*) chains and the IL-4 receptor subunits are presented with interacting residues on the IL-4 Stapler Fab show as sticks and labeled. Below, two-dimensional interaction maps between amino acids on IL-4 Stapler V_H (magenta), IL-4 Stapler V_L (olive), IL-4Rα (blue), and γ_c (yellow) are provided for each of the four IL-4 Stapler variable domain/IL-4 receptor subunit interfaces. Interactions between side chains are represented as lines whereas interactions between side chains and backbone are shown as arrows with the arrowheads pointing to the backbone. Black lines represent Van der Waals and hydrophobic contacts and red lines denote hydrogen bonds or electrostatic interactions.

Figure S8

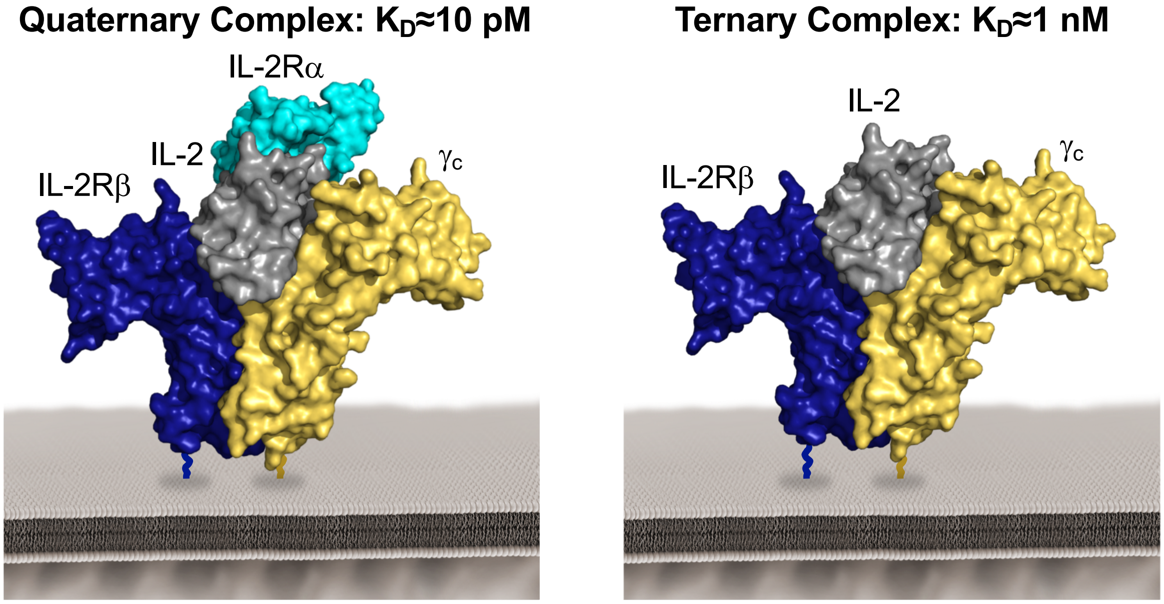

IL-2 cytokine-receptor quaternary and ternary complex structures.
Crystal structures of the quaternary (*left*) and ternary (*right*) IL-2 cytokine-receptor complexes. The quaternary complex consists of the IL-2 cytokine and the IL-2Rα (cyan), IL-2Rβ (navy), and γ_c (yellow) subunits, whereas the ternary complex lacks the IL-2Rα subunit. Note that the quaternary complex has 100-fold stronger affinity than the ternary complex.

Figure S9

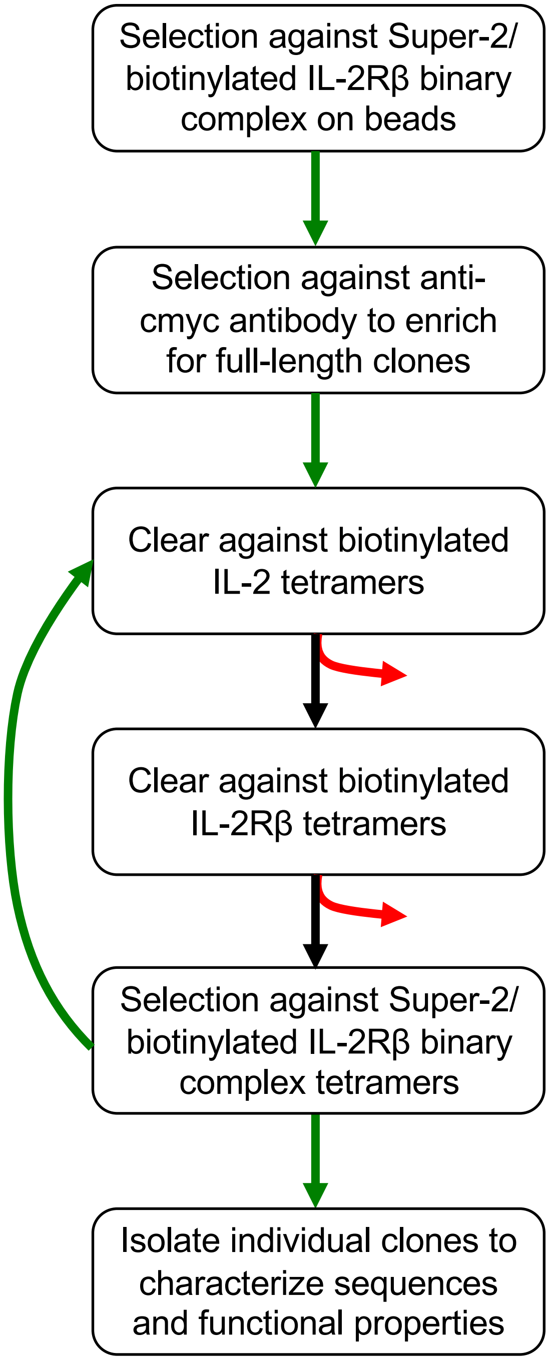

IL-2 binary complex stapler selection strategy.
Flow diagram depicting the selection strategy implemented on a naïve human non-immune yeast-displayed scFv library to evolve antibody fragments that stabilize IL-2/IL-2Rβ binary complex formation. Note the use of Super-2 in place of the IL-2 cytokine to stabilize the target complex.

Figure S10

IL-2 Binary Complex Stapler CDR_3 sequences:

*Positions that were randomized in the affinity maturation library are indicated in red.

	CDR_3 V_H												CDR_3 V_L											
	101	102	103	104	105	106	107	108	109	110	111	112	233	234	235	236	237	238	239	240	241	242	243	244
IL-2B Stapler	C	A	R	A	V	S	G	T	F	D	Y	W	C	Q	S	Y	D	D	N	Y	H	A	V	F
amIL-2B Stapler	C	A	R	A	T	Y	G	T	Y	D	Y	W	C	Q	S	Y	D	Q	N	Y	H	A	V	F

Affinity Maturation Library Design:

	104	105	106	107	108	109	236	237	238	239	240
Wild Type	A	V	S	G	T	F	Y	D	D	N	Y
Library Design	A/I/M/ T/V	A/I/M/ T/V	A/D/N/ S/T/Y	A/G/I/ S/T/V	A/D/N/ S/T/Y	F/H/I/ L/N/Y	F/I/N/Y	D/E/H/ K/N/Q	D/E/H/ K/N/Q	D/E/H/ K/N/Q	F/I/N/Y

IL-2 binary complex stapler variable domain sequences and affinity maturation library design. V_H and V_L CDR3 region sequences for the IL-2 binary complex stapler (IL-2B Stapler) and its affinity matured variant, amIL-2B Stapler (top). Allowed amino acids at each of the eleven positions that were mutagenized in the yeast-displayed library used to select the affinity matured stapler variant are presented in the bottom table.

Figure S11

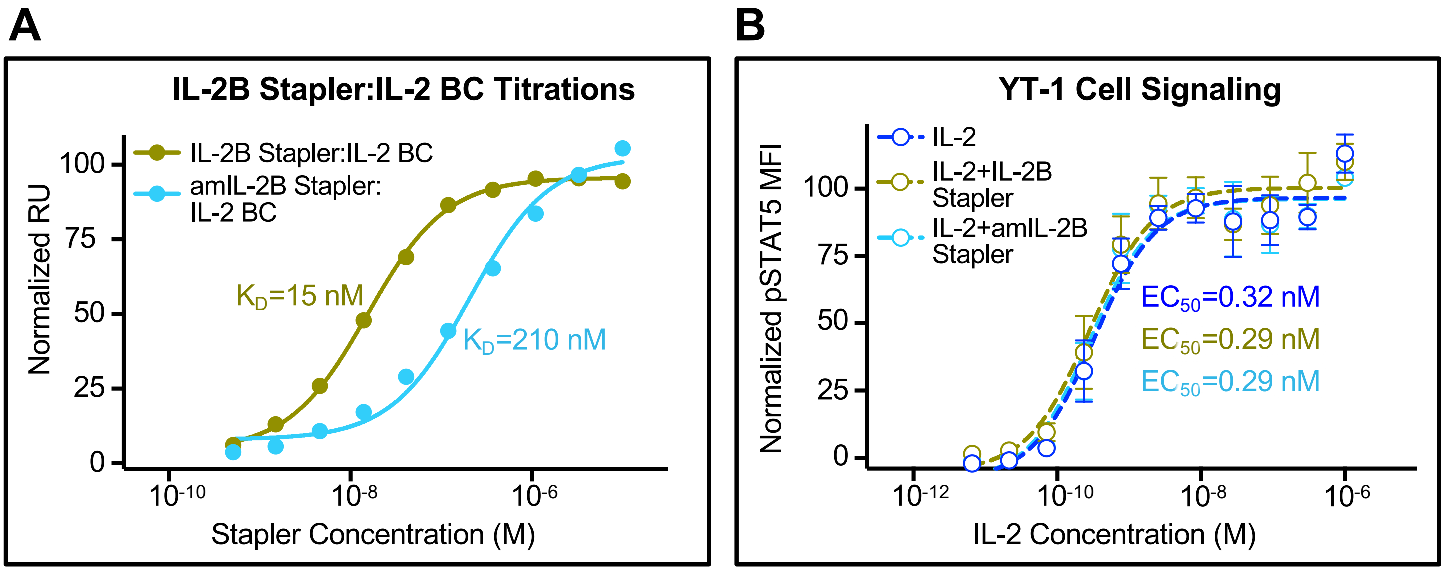

IL-2 binary complex staplers do not enhance signaling potency of the native cytokine.
(**A**) Comparison of the equilibrium surface plasmon resonance titrations of IL-2 BC binding to IL-2B Stapler versus amIL-2B Stapler. K_D values were calculated by fitting equilibrium titration curves to a logistic model via non-linear regression. (**B**) Comparison of YT-1 human NK cell STAT5 phosphorylation responses to IL-2 treatment without scFv present (unfilled blue circles) and with saturating concentrations of either IL-2B Stapler (unfilled gold circles) or amIL-2B Stapler (unfilled cyan circles). EC_{50} values are indicated. Error bars indicate SD.
